# Current Perspective on the Membrane-Damaging Action of Thermostable Direct Hemolysin, an Atypical Bacterial Pore-forming Toxin

**DOI:** 10.3389/fmolb.2021.717147

**Published:** 2021-07-23

**Authors:** Pratima Verma, Kausik Chattopadhyay

**Affiliations:** Department of Biological Sciences, Indian Institute of Science Education and Research Mohali, Mohali, India

**Keywords:** pore-forming toxin, thermostable direct hemolysin, membranes, actinoporins, oligomer

## Abstract

Thermostable direct hemolysin (TDH) is the major virulence determinant of the gastroenteric bacterial pathogen *Vibrio parahaemolyticus*. TDH is a membrane-damaging pore-forming toxin (PFT). TDH shares remarkable structural similarity with the actinoporin family of eukaryotic PFTs produced by the sea anemones. Unlike most of the PFTs, it exists as tetramer in solution, and such assembly state is crucial for its functionality. Although the structure of the tetrameric assembly of TDH in solution is known, membrane pore structure is not available yet. Also, the specific membrane-interaction mechanisms of TDH, and the exact role of any receptor(s) in such process, still remain unclear. In this mini review, we discuss some of the unique structural and physicochemical properties of TDH, and their implications for the membrane-damaging action of the toxin. We also present our current understanding regarding the membrane pore-formation mechanism of this atypical bacterial PFT.

## Introduction

Pore-forming toxins (PFTs) are the unique class of proteins that damage cell membranes by forming pores in the membrane lipid bilayer (Mondal et al., 2018). Pore-formation in the cell membranes allows unregulated flow of ions, water, and other molecules that, in turn, perturb the cellular homeostasis and ultimately lead to cell death (Mondal et al., 2020; Verma et al., 2021). Molecular architectures of the PFTs are designed in such a specialised manner that allow them to be compatible with two distinct physicochemical environments. PFTs are generally secreted as water-soluble molecules, which upon encountering the target cell membranes undergo structural and organizational changes, assemble into transmembrane oligomeric pores, and get anchored into the amphipathic milieu of the membrane lipid bilayer. Owing to such property, PFTs are aptly designated as a unique class of dimorphic proteins (Heuck et al., 2001). Based on the secondary structures of the membrane-spanning regions, PFTs are broadly classified either as the *α*-PFTs or *β*-PFTs (Mondal and Chattopadhyay, 2020). However, it is important to emphasize that the PFTs display remarkable diversity in their sequences, structures, and mechanism of action.

PFTs are ubiquitously found in all the kingdoms of life. Notably, bacterial PFTs constitute the largest class of bacterial protein toxins, and play significant roles in their virulence mechanisms (Verma et al., 2021). Thermostable direct hemolysin (TDH) is one such PFT secreted by the Gram-negative bacterial pathogen *Vibrio parahaemolyticus* that is recognised as one of the major causative agents of the seafood-borne acute gastroenteritis (Wang et al., 2015; Cai and Zhang, 2018). Most of the clinical isolates of *V. parahaemolyticus* exhibit a characteristic hemolysis ring pattern, the so called Kanagawa phenomenon, on the Wagatsuma blood agar. This Kanagawa phenomenon is attributed to the pore-forming activity of TDH (Wang et al., 2015). The gene encoding TDH is found in most of the clinical isolates of *V. parahaemolyticus*, and is also present in some of the non-clinical strains of the bacterium (Raghunath, 2014). TDH-related proteins are also documented in other *Vibrio* species such as *V. cholerae*, *V. mimicus,* and *V. hollisae* (Raghunath, 2014)*. V. parahaemolyticus* TDH has been characterized as a membrane-damaging PFT. It has been shown to exert several pathophysiological effects such as hemolytic activity, cytotoxicity, enterotoxicity, and cardiotoxicity (Sakurai et al., 1975; Honda et al., 1976; Raimondi et al., 2000). Based on such properties, TDH is considered as the major virulence factor of *V. parahaemolyticus*.

Here, we present a brief overview of our current understanding regarding the structure-function mechanisms in the membrane-damaging action of TDH. We also highlight the existing lacuna in our knowledge about the mechanism of membrane pore-formation by this atypical bacterial PFT.

## Secretion and Regulation of TDH

*V. parahaemolyticus* harbours two sets of *tdh* gene (*tdh1* and *tdh2*) present in the 80 kb pathogenicity island, Vp-PAI. The genes encoding TDH across various strains are well-conserved and their gene products are also immunologically identical (Nishibuchi and Kaper, 1990; Nishibuchi and Kaper, 1995). The *tdh* gene is under the tight regulation of various transcriptional regulators as well as environmental factors. The expression of the *tdh* gene is under the regulation of the *toxRS* operon that works in a growth medium-dependent manner (Lin et al., 1993). Two ToxR-like regulatory proteins, VtrA and VtrB control the expression of around 60 genes in the Vp-PAI region, including positive regulation of TDH (Kodama et al., 2010). In addition, ToxR mediates the activation of CalR, a LysR-type transcription regulator that represses *tdh2* transcription, by inhibiting ToxR binding to the *tdh2* promoter region. In a feedback loop manner, CalR also represses *toxR* and its own expression (Zhang et al., 2017). Another study has shown that the *luxM/luxS-*dependent quorum sensing mediates the regulation of the hemolytic activity along with biofilm formation in *V. parahaemolyticus. luxM* downregulates *tdh* gene expression, and therefore compromises the hemolytic activity, while *luxS* upregulates the expression of the hemolysin (Guo et al., 2018). Also, HN-S (nucleoid-associated DNA binding regulator) and Hfq (a global transcription regulator) have been shown to repress the production of TDH (Nakano et al., 2008; Sun et al., 2014). Growth environments such as iron-deficient conditions, conjugated bile salts and altered temperature conditions have also been shown to influence the production of TDH (Cai and Zhang, 2018).

The precursor form of TDH is composed of 189 amino acid residues including a 24 amino acids-long N-terminal signal peptide. TDH takes unconventional routes for its secretion from the bacterial cells*.* Owing to the presence of a signal peptide, TDH is secreted through the type-2 secretion system as the mature exotoxin. In addition, TDH is also released as an effector protein through the type-3 secretion system. Mature TDH can be transported back to the cytoplasm through the periplasmic space, and then gets recruited to the type-3 secretion system 2 for secretion as an effector molecule (Matsuda et al., 2019). Functional implications of both these mode of secretion appear to be distinct. It has been shown that TDH, as effector molecule, can lead to the intestinal fluid accumulation in the rabbit model. In contrast, TDH as exotoxin has been shown to cause intra-peritoneal lethality in the murine model (Matsuda et al., 2019).

## Structure and Physicochemical Properties of TDH

**FIGURE 1:**
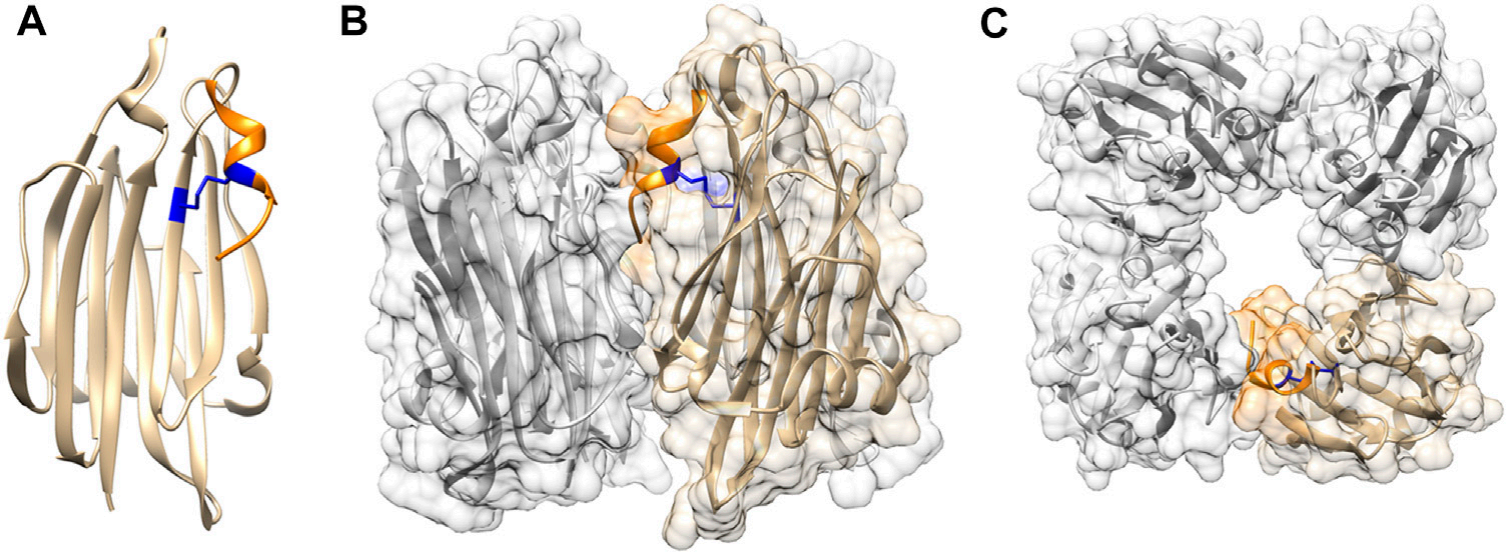
Structural features of TDH **(A)** TDH protomer adopts a core *β*-sandwich domain. C-terminal region (shown in orange) is attached to the core domain through intra-protomer disulphide linkage (shown in blue) **(B)** Side view of tetrameric assembly of TDH. One of the protomers is highlighted in colour as referred in panel A **(C)** Top view of the TDH tetrameric assembly showing the central pore. Structural models of TDH were generated with UCSF Chimera using the structural coordinate obtained from the protein data bank (PDB; PDB ID 3A57).

Crystal structure of TDH at 1.5 Å resolution has shown that the 165 residue-long mature form of the toxin adopts a *β*-sandwich core domain (Yanagihara et al., 2010)(Figure 1A). Notably, such a fold is not commonly documented in the bacterial PFTs. The *β*-sandwich core domain of the TDH monomer is composed of 10 *β*-strands packed in two layers, and is flanked by a flexible eleven residue-long stretch at the N-terminus that could not be modelled in the crystal structure, and a nine residue-long region at the C-terminal boundary. Crystal structure of TDH also depicts a unique tetrameric assembly of the toxin that has a 23 Å-diameter central pore with a depth of 50 Å (Figures 1B, C). Tetramer formation by TDH in solution has also been confirmed by analytical ultracentrifugation-based studies (Hamada et al., 2007). Tetrameric assembly of TDH is crucial for its membrane-damaging hemolytic activity. Disruption of the inter-protomer interactions that block tetramer formation has been shown to compromise the membrane-damaging action of TDH (Yanagihara et al., 2010; Kundu et al., 2017).

Another unique physicochemical property of TDH is that it exhibits a temperature-dependent reversible amyloidogenic behaviour (Fukui et al., 2005). TDH tends to form amyloid-like structures upon heating at 60°C, and loses its membrane-damaging hemolytic activity. However, upon further heating at 90°C, it reverts back into the functionally active form. This phenomenon is designated as the Arrhenius effect, and has been observed in case of certain other PFTs from *Staphylococcus aureus, Bacillus cereus, Klebsiella pneumoniae* and *Pseudomonas aeruginosa* (Fukui et al., 2005)*.* Physiological significance of such reversible amyloidogenic property of TDH remains unclear at present.

## Structural Similarity of TDH to the Eukaryotic PFTs in the Actinoporin Family

Based on the structural similarity, TDH finds its nearest neighbours in the actinoporin family of eukaryotic PFTs that include Equinatoxin II, Fragaceatoxin C and Sticholysin II (Yanagihara et al., 2010; Caaveiro and Tsumoto, 2021). The actinoporins constitute a unique family of eukaryotic *⍺*-PFTs secreted by the sea anemones (Ramírez-Carreto et al., 2020). As observed with TDH, actinoporins are also generally produced as water-soluble, single domain protein with a *β*-sandwich core architecture (Rojko et al., 2016). Such similarity in the structures suggests possible evolutionary connection between TDH and actinoporins.

In spite of sharing an overall similar core *β*-sandwich domain architecture, TDH structure highlights a number of unique features that are not observed in the actinoporins. For example, TDH possesses two cysteine residues (Cys151 and Cys161), and an intra-chain disulphide bond is formed between these two residues (Figure 1A). Cys161 is located within the 9 residue-long extension at the C-terminal boundary of the *β*-sandwich domain. As a result, Cys151-Cys161 disulphide linkage acts to hold this C-terminal extension against the core *β*-sandwich domain (Kundu et al., 2017). Interestingly, actinoporins are generally cysteine-less proteins (Ramírez-Carreto et al., 2020). Moreover, they do not possess the extended C-terminal region that is present in TDH (Kundu et al., 2017). In contrast, the region at the N-terminal side of the *β*-sandwich domain of the actinoporins is considerably longer (∼thirty residue long), and adopts *α*-helical structure (Carretero et al., 2018). Notably, the N-terminal region of TDH is only eleven residue-long, and it appears to be highly flexible, and is not resolved in the crystal structure (Yanagihara et al., 2010; Kundu et al., 2020). Another notable difference between the structures of TDH and actinoporins is the assembly state in solution. Unlike TDH, actinoporins generally remain as monomer in solution, and upon encountering the target membranes they assemble into the oligomeric pores (Rojko et al., 2016). In contrast, TDH remains as tetramer in solution, even before binding to the target membranes (Yanagihara et al., 2010). Such differences in the assembly states may have crucial implications for the membrane-damaging pore-formation mechanisms employed by TDH and the actinoporin family of PFTs.

Altogether, TDH shows remarkable similarity to the actinoporins in terms of harbouring the central *β*-sandwich scaffold. Yet, at the same time, TDH highlights the presence of distinct structural features. It remains unclear at present how the structure-function mechanisms have evolved in TDH and the actinoporins that are produced by the organisms from two completely distinct kingdoms of life.

## C-Terminal Region of TDH

As described above, analysis of the TDH sequence and structure shows presence of an extended nine residue-long stretch (^157^SFFECKHQQ^165^) at the C-terminal end of the *β*-sandwich domain. Part of this region, ^157^SFFECK^162^, adopts a 3_10_ -helix structure, while the last three residues (^163^HQQ^165^) do not appear to form any secondary structure (Figure 1). In the tetrameric assembly of TDH, the C-terminal region of each of the protomers is positioned at the inter-protomer interface. Moreover, the residue Gln164 within the C-terminal region of one protomer is engaged in interaction(s) with the neighbouring protomer (Yanagihara et al., 2010). Consistent with such observations, deletion of the C-terminal region as well as mutation of Gln164 have been shown to compromise tetramer formation and membrane-damaging activity of TDH (Kundu et al., 2017). This in turn suggests that the extended C-terminal region of TDH plays a pivotal role in stabilizing the tetrameric assembly state that is crucial for the biological functionality of the toxin.

As mentioned above, Cys161 located within the C-terminal region of TDH forms a disulphide linkage with Cys151 in one of the *β*-strands of the *β*-sandwich domain. Thus, the Csy151-Cys161 disulphide linkage acts to lock the C-terminal region against the core *β*-sandwich domain. Moreover, this disulphide bond also possibly helps to maintain an appropriate structural disposition of the C-terminal region at the inter-protomer interface, so that it can establish the interactions with the neighbouring protomer for stabilizing the tetrameric assembly of TDH (Kundu et al., 2017). Altogether, such a restrained configuration of the C-terminal region, mediated by the disulphide bond, acts as a regulatory mechanism for the unique tetrameric assembly of TDH, and is not generally documented in the PFT family.

## N-Terminal Region of TDH

The mature form of TDH contains a stretch of eleven residues (^1^FELPSVPFPAP^11^) at the N-terminal side of the core *β*-sandwich domain. This N-terminal region is not visible in the crystal structure. It has been argued that this motif is possibly having high degree of conformational fluctuation, as the result of which the electron density corresponding to this region could not be resolved (Yanagihara et al., 2010). Analysis of the TDH amino acid sequence also predicts this N-terminal eleven residue-long stretch to be highly disordered. It has been shown that the presence of the N-terminal region promotes amyloidogenic propensity of TDH when exposed to the elevated temperature of 60°C (Kundu et al., 2020). Physiological implication of the observed amyloidogenic propensity of TDH displayed at the elevated temperature remains unclear at present. Another distinct feature of this eleven residue N-terminal region of TDH is that it is populated with aromatic and hydrophobic residues, along with four proline residues. Presence of such a large number of proline residues possibly explains why this motif is having an increased disorder and higher conformational fluctuations. It has been shown in a recent study that the deletion and mutation of the N-terminal region of TDH result into abrogation of the membrane-binding ability and drastic reduction in the membrane-damaging cell-killing activity. Moreover, toward restraining the predicted conformational fluctuation of the N-terminal region, covalent locking of this motif against the central *β*-sandwich domain, through introduction of the engineered disulphide bond, also results into compromised membrane-damaging functionality of TDH (Kundu et al., 2020). All these observations clearly suggest that the presence of the eleven residue-long N-terminal motif, as well as it’s flexible structural disposition are indispensable for the membrane-damaging activity of TDH. However, it still remains an enigma how exactly this motif contributes to the TDH functionality.

## Pore-formation Mechanism of TDH

Archetypical members of the PFT family generally follow an overall similar mechanism of membrane pore formation. It generally involves the following distinct steps: 1) water-soluble monomers of the PFT bind to the target membranes; 2) membrane-bound toxin monomers associate with each other to form the oligomeric assembly; 3) Structural rearrangement and membrane-insertion of the pore-forming motifs from the toxin protomers create the transmembrane scaffold (α-helical bundle or *β*-barrel) of the pore (Dal Peraro and van der Goot, 2016; Mondal et al., 2020).

### Membrane-Binding Process

Interaction of a PFT with the target cell membranes is the first crucial step in its membrane-damaging action. However, the membrane-interaction process of TDH remains unclear at present. Disparate cell types display varying level of susceptibility in response to TDH, presumably due to differential extent of toxin binding to the cells (Sakurai et al., 1976; Cai and Zhang, 2018). Whatsoever, requirement of any specific cell-surface receptor(s), if any, and exact identity of such receptor(s) for TDH remain to be established. In one of the studies, GT1 ganglioside has been considered as a putative receptor for TDH (Takeda et al., 1976). In another report, asialo-GM2 ganglioside has also been proposed as a possible receptor (Douet et al., 1996). However, it still remains uncertain whether these gangliosides indeed act as the cellular receptor(s) for TDH.

Some of the PFTs display direct interactions with membrane lipid components. For example, actinoporin family of PFTs, that are structurally similar to TDH, display ability to recognise and bind to the phosphocholine moiety of phosphatidylcholine and/or sphingomyelin (Cosentino et al., 2016; Ramírez-Carreto et al., 2020). Moreover, actinoporin structures harbour distinct binding site(s)/pocket(s) involving conserved set of residues for lipid binding (Rojko et al., 2016). Although similar motif(s) could be observed on the surface of the TDH structure, any specific lipid-binding propensity of TDH remains obscure at present.

In one study, TDH has been shown to associate with the detergent-resistant membrane fractions or lipid raft-like regions of the target nucleated cells (Matsuda et al., 2010). Disruption of the raft-like detergent-resistant membrane fractions *via* depletion of membrane cholesterol has also been found to inhibit cytotoxicity of TDH against these nucleated cells. Depletion of sphingomyelin from the cells has also been shown to compromise the association of TDH with the detergent-resistant membrane fractions, and cytotoxicity. However, any direct interaction of TDH with membrane sphingomyelin and cholesterol could not be established. It is also interesting to note that the depletion of membrane cholesterol could not compromise pore-forming hemolytic activity of TDH against the erythrocytes (Matsuda et al., 2010). Altogether, roles of cholesterol and sphingomyelin, if any, in the mode of action of TDH remain enigmatic, and need to be explored in detail.

### Mechanism of Pore-formation

Structural basis of membrane pore-formation by TDH also remains obscure at present. Although the crystal structure has been determined for the water-soluble tetrameric form of the toxin, structure of the membrane-bound pore state remains unknown till date. Unlike the prototype PFTs, TDH remains as pre-formed tetramers in solution prior to its interaction with the target membranes. Crystal structure of the water-soluble TDH tetramer shows that it has a central pore (Yanagihara et al., 2010). However, it remains unknown how this assembly state would create a water-filled transmembrane scaffold upon associating the membrane lipid bilayer. Exact identity of the pore-forming motif of TDH that could create the transmembrane region remains unclear. In the case of the actinoporins such as Fragaceatoxin C, it has been shown that the thirty amino acid-long N-terminal *α*-helical region acts as the pore-forming motif, and passes through the membrane lipid bilayer (Tanaka et al., 2015). Although TDH shares overall structural similarity to the Fragaceatoxin C, N-terminal region of TDH is only eleven residue-long, and therefore it would presumably be considerably shorter for traversing the entire depth of the membranes. Nevertheless, the presence of the N-terminal region of TDH, along with its flexible disposition, has been shown to be crucial for the membrane-damaging function, thus implicating its potential role in the pore-formation mechanism (Kundu et al., 2020).

**FIGURE 2:**
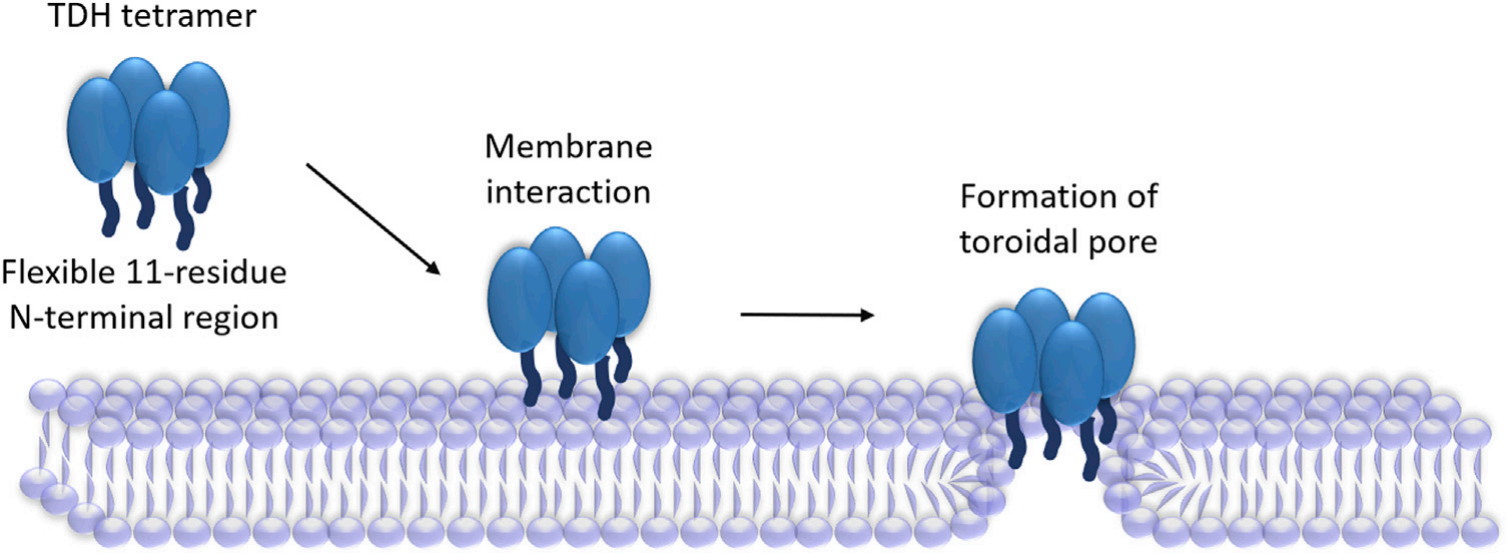
Proposed mechanism of pore-formation by TDH. TDH tetramer interacts with the target membranes. N-terminal regions (shown as dark blue extensions) of the TDH protomers play critical roles in the pore-formation mechanism. TDH may form toroidal pores, in which pore-lining could be created with the collaboration of the N-terminal regions of the protein and membrane lipids.

Archetypical PFTs generally form “protein-only” pores, in which the transmembrane scaffold is constituted from the pore-forming motif of the protein (Gilbert et al., 2014). However, a distinct ‘toroidal pore’ model of pore-formation has now emerged in the recent years, where the pore lining is formed by the collaborative contribution of the protein motif(s) and the membrane lipids (Gilbert et al., 2014). In this model, the protein motif(s) generally tend to distort the arrangement of the membrane phospholipids in such a manner that would create the membrane perforation. This, in turn, precludes the requirement of the pore-forming motif(s) of the PFT to traverse the entire depth of the plasma membranes. Indeed, a “toroidal pore” model of pore-formation has been suggested for the actinoporin family members, Equinatoxin II and Sticholysin II (Malovrh et al., 2003; Rojko et al., 2016). Therefore, the N-terminal region of TDH, in spite of its shorter length, can possibly contribute for the toroidal pore formation (Figure 2). However, involvement of any other structural motif of TDH in the pore-formation process cannot be ruled out at present. Determination of the high-resolution structure of the membrane-associated pore state of TDH would possibly provide conclusive answer to these questions.

## Pathophysiological Functionalities of TDH

Being a potent PFT, TDH causes membrane damage that in turn can lead to the ion imbalance in the target cells. TDH has been shown to induce Ca^2+^ influx in its target cells. However, the cytotoxicity induced by TDH has been suggested to be independent of any Ca^2+^-dependent pathway (Tang et al., 1995). TDH has also been shown to trigger Cl^−^ secretion from the target colonic epithelial cells, and the process appears to be dependent on the elevated cytosolic Ca^2+^ levels (Takahashi et al., 2000). TDH exhibits cytotoxicity along with morphological changes and several pathophysiological effects against a variety of cell types (Sakurai et al., 1976). Apart from cytotoxicity, TDH is also capable of triggering variety of other pathophysiological responses such as cardiotoxicity and enterotoxicity (Honda et al., 1976; Raimondi et al., 2000). TDH has been shown to induce apoptosis-like features in the target cells (Naim et al., 2001). Moreover, TDH is capable of eliciting the host immune responses by inducing the activation of caspase-1 *via* NLRP3 inflammosome activation (Higa et al., 2013). Whatsoever, it is important to appreciate that in addition to all these activities, TDH may also evoke a variety of other cellular responses that are yet to be deciphered.

## Conclusion

Over the years, various structural studies on TDH have highlighted critical molecular details about this membrane-damaging PFT. Available crystal structure has provided valuable insights regarding its structure-function relationship. It has revealed the unique structural fold adopted by TDH that has a striking resemblance to those of the actinoporin family of eukaryotic PFTs. Also, the unique tetrameric assembly of TDH is proven to be essential for its functionality. These studies have shaped our current understanding regarding the structural basis of the membrane-damaging action of TDH. However, the exact mechanism of membrane pore-formation by TDH is yet to be elucidated. Moreover, the membrane pore structure of TDH is not yet available. Future studies addressing such issues would not only provide valuable insights regarding the mode of action of TDH, but would also shed light into the evolution of the structure-function paradigm in the structurally-related PFTs of bacterial and eukaryotic origin.

## Abbreviations

PFT, Pore-forming toxin; TDH, thermostable direct hemolysin.
